# Characterising personal, household, and community PM_2.5_ exposure in one urban and two rural communities in China

**DOI:** 10.1016/j.scitotenv.2023.166647

**Published:** 2023-12-15

**Authors:** Ka Hung Chan, Xi Xia, Cong Liu, Haidong Kan, Aiden Doherty, Steve Hung Lam Yim, Neil Wright, Christiana Kartsonaki, Xiaoming Yang, Rebecca Stevens, Xiaoyu Chang, Dianjianyi Sun, Canqing Yu, Jun Lv, Liming Li, Kin-Fai Ho, Kin Bong Hubert Lam, Zhengming Chen, China Kadoorie Biobank collaborative group

**Affiliations:** a Clinical Trial Service Unit and Epidemiological Studies Unit, Nuffield Department of Population Health, University of Oxford, UK; b Oxford British Heart Foundation Centre of Research Excellence, University of Oxford, UK; c School of Public Health, Xi'an Jiaotong University, China; d The Jockey Club School of Public Health and Primary Care, The Chinese University of Hong Kong, Hong Kong; e School of Public Health, Key Lab of Public Health Safety of the ministry of Education and NHC Key Lab of Health Technology Assessment, Fudan University, China; f Big Data Institute, Li Ka Shing Centre for Health Information and Discovery, University of Oxford, UK; g National Institute of Health Research Oxford Biomedical Research Centre, Oxford University Hospitals NHS Foundation Trust, John Radcliffe Hospital, UK; h Asian School of the Environment, Nanyang Technological University, Singapore; i Lee Kong Chian School of Medicine, Nanyang Technological University, Singapore; j Earth Observatory of Singapore, Nanyang Technological University, Singapore; k MRC Population Health Research Unit, Nuffield Department of Population Health, University of Oxford, UK; l NCDs Prevention and Control Department, Sichuan CDC, China; m Department of Epidemiology and Biostatistics, School of Public Health, Peking University Health Science Center, China; n Peking University Center for Public Health and Epidemic Preparedness and Response, China

**Keywords:** Fine particulate matter, Exposure assessment, Wearable sensor, Solid fuels, Cooking, Heating

## Abstract

**Background:**

Cooking and heating in households contribute importantly to air pollution exposure worldwide. However, there is insufficient investigation of measured fine particulate matter (PM_2.5_) exposure levels, variability, seasonality, and inter-spatial dynamics associated with these behaviours.

**Methods:**

We undertook parallel measurements of personal, household (kitchen and living room), and community PM_2.5_ in summer (May–September 2017) and winter (November 2017-Janauary 2018) in 477 participants from one urban and two rural communities in China. After stringent data cleaning, there were 67,326–80,980 person-hours (n_total_ = 441; n_summer_ = 384; n_winter_ = 364; 307 had repeated PM_2.5_ data in both seasons) of processed data per microenvironment. Age- and sex-adjusted geometric means of PM_2.5_ were calculated by key participant characteristics, overall and by season. Spearman correlation coefficients between PM_2.5_ levels across different microenvironments were computed.

**Findings:**

Overall, 26.4 % reported use of solid fuel for both cooking and heating. Solid fuel users had 92 % higher personal and kitchen 24-h average PM_2.5_ exposure than clean fuel users. Similarly, they also had a greater increase (83 % vs 26 %) in personal and household PM_2.5_ from summer to winter, whereas community levels of PM_2.5_ were 2–4 times higher in winter across different fuel categories. Compared with clean fuel users, solid fuel users had markedly higher weighted annual average PM_2.5_ exposure at personal (78.2 [95 % CI 71.6–85.3] μg/m^3^ vs 41.6 [37.3–46.5] μg/m^3^), kitchen (102.4 [90.4–116.0] μg/m^3^ vs 52.3 [44.8–61.2] μg/m^3^) and living room (62.1 [57.3–67.3] μg/m^3^ vs 41.0 [37.1–45.3] μg/m^3^) microenvironments. There was a remarkable diurnal variability in PM_2.5_ exposure among the participants, with 5-min moving average from 10 μg/m^3^ to 700–1200 μg/m^3^ across different microenvironments. Personal PM_2.5_ was moderately correlated with living room (Spearman r: 0.64–0.66) and kitchen (0.52–0.59) levels, but only weakly correlated with community levels, especially in summer (0.15–0.34) and among solid fuel users (0.11–0.31).

**Conclusion:**

Solid fuel use for cooking and heating was associated with substantially higher personal and household PM_2.5_ exposure than clean fuel users. Household PM_2.5_ appeared a better proxy of personal exposure than community PM_2.5_.

## Highlights

• We assessed personal, household, and community level PM_2.5_ in 1 urban and 2 rural regions in China.
• We estimated adjusted seasonal and annual mean PM_2.5_ levels by key characteristics (e.g. fuel use).
• Solid fuel use for cooking and heating were related to markedly higher personal and household PM_2.5_ exposure.
• Solid fuel users had >15 times higher annual PM_2.5_ exposure than the WHO Air Quality Guideline level.
• Household PM_2.5_ was a better proxy of personal exposure than community PM_2.5_, especially in solid fuel users.

## Graphical abstract

**Figure:**
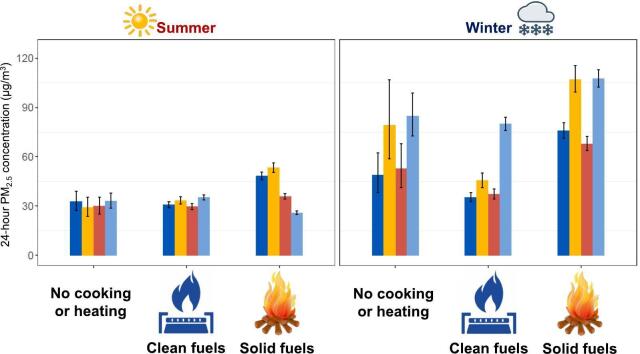

## 1 Introduction

The continued reliance on fossil fuels to meet the growing energy demand from a rapidly urbanised population, combined with limited and inadequate regulatory regimes for environmental protection and poor enforcement, have worsened ambient air pollution in many low- and middle-income countries (LMICs). Additionally, about 3 billion individuals still rely on solid fuels (e.g. coal, wood) for cooking and heating, leading to intensive household air pollution (Health Effect Institute, 2020; Stoner et al., 2021). Fine particulate matter (PM_2.5_) from domestic use of solid fuels and ambient sources together constitute the top environmental risk factor of disease burden globally, estimated to account for >6 million premature deaths in 2019 (Health Effect Institute, 2020). Despite the global health significance, substantial uncertainties persist in understanding the exposure-disease relationships and, subsequently, disease burden estimation. This is mainly due to the reliance on exposure proxies, namely modelled ambient air pollution levels around residential addresses and self-reported fuel use for indoor or household air pollution exposure in most existing epidemiological studies (Chan et al., 2020; Morawska et al., 2018).

Until recently, directly measured air pollution exposure data were uncommon in large (n > 100,000) population-based epidemiological studies. Most measurement studies had relatively small sample sizes, assessed primarily kitchen PM_2.5_ levels, and focused on rural communities (Balakrishnan et al., 2018; Benka-Coker et al., 2020; Ni et al., 2016; Shupler et al., 2018; Shupler et al., 2020; Sidhu et al., 2017; Snider et al., 2018; Tong et al., 2018; Ye et al., 2020). The largest relevant study to date (PURE-Air) collected 48-hour aggregated kitchen and personal PM_2.5_ data in 2365 households and 910 individuals, respectively, in rural areas from eight LMICs (Shupler et al., 2020; Shupler et al., 2022). They found substantial variability in kitchen and personal PM_2.5_ levels by cooking fuel types and across countries, with solid fuel users tending to show significantly higher exposure (Shupler et al., 2020; Shupler et al., 2022). However, due to the relatively short measurement window, limited repeated seasonal measurements, and inadequate coverage of heating season exposure, there remains ambiguity regarding both within-week and seasonal exposure variability within and between individuals. There is also a need of time-resolved data and parallel assessment of not only personal and kitchen PM_2.5_ but also living room and ambient levels to better understand the spatial-temporal dynamics of PM_2.5_ exposure. Data from urban areas will also offer additional insights into the urban-rural contrast in PM_2.5_ exposure patterns.

Here we report detailed analyses of questionnaire data on personal characteristics, fuel use, and time-resolved PM_2.5_ exposure data at personal, household, and community levels from one urban and two rural areas in the China Kadoorie Biobank (CKB), across the warm and cool seasons (Chan et al., 2021). The present report aims to i) examine both aggregated and time-resolved PM_2.5_ levels by fuel use and other key characteristics; and ii) clarify personal-household-community gradient of PM_2.5_ exposure.

## 2 Materials and methods

### 2.1 Study design and sample

CKB is an ongoing prospective cohort study of ~512,000 adults aged 30–79 years recruited from ten diverse areas of China during 2004–2008 (Chen et al., 2005; Chen et al., 2011). The CKB-Air study was nested within CKB, and details of the design, data collection procedures, data cleaning and processing, and participant characteristics have been published previously (Chan et al., 2021). Briefly, 477 participants (mean age 58 years, 72 % women) were recruited via convenient sampling from two rural (Gansu, Henan) and one urban (Suzhou) CKB study sites (eFigure 1), selected to capture a diverse range of fuel use patterns (Chan et al., 2017). The study involved repeated assessment of air pollution and time-activity in the warm (May–September 2017; hereafter referred to as ‘summer’) and cool (November 2017–January 2018; ‘winter’) seasons, with a household questionnaire on participant characteristics and usual fuel use patterns administered in the winter (Chan et al., 2021). In particular, 452 and 450 individuals participated in the summer and winter assessments, respectively (37 participants were not available in winter and were replaced by 35 other eligible CKB participants in the same community). The participants included in the two seasons were similar in their socio-demographics and lifestyle characteristics documented in 2004–2008 during the baseline assessment (Chan et al., 2021).

The study was approved by the Oxford University Tropical Research Ethics Committee, Oxford, UK (Ref: 5109-17) and the institutional review board of Fuwai Hospital, Chinese Academy of Medical Sciences, Beijing, China (Ref: 2018-1038). All participants provided written informed consent upon recruitment.

### 2.2 Questionnaire data

Trained health workers administered a laptop-based household questionnaire in the cool season, to assess personal characteristics (age, sex, household income, occupation, active and passive smoking) and exposure to household air pollution (cooking and heating patterns and all fuel types used) (Text S2). For those who reported different cooking patterns or fuel use in summer, additional questions on exposure during summer were asked. While many previous studies focused on a single primary cooking or heating fuels, we attempted to capture ‘fuel stacking’ by assessing all fuel types used (Chan et al., 2017). Cooking fuel combinations were derived based on all fuel types reported to be ‘used in most meals’ or ‘sometimes’ (among those who did not report any fuel ‘used in most meals’). A similar approach was undertaken to derive ‘heating fuel combination’ based on the duration of heating fuel use during winter. Clean fuels include gas, electricity, solar, and city-wide district heating (for heating only); solid fuels include coal (smoky/smokeless), coal briquette, charcoal, wood, and crop residue. The electronic questionnaire have built-in error and logic checks to minimise missing data and human errors.

### 2.3 Air pollution data

#### 2.3.1 Air pollution monitors

The study aimed to obtain 120 consecutive hours (from Thursday to Tuesday, covering weekdays and weekend) of measurements (at 1-min resolution) of fine particulate matter (PM_2.5;_ μg/m^3^) levels, temperature, and relative humidity (%) in three different microenvironments (personal, kitchen, and living room) for each participant, covering both summer and winter except for those who only participated in one season (n = 72). The measurements were taken using PATS (Particle and Temperature Sensor; Berkeley Air Monitoring Group, CA, USA), an internationally validated low-cost nephelometer-based device (R^2^ range: 0.90–0.99 with reference to both with well-established time-resolved instruments [e.g. TSI DustTrak] and gravimetric measurements) developed for high household air pollution settings (PM_2.5_ detection range: 10–30,000 μg/m^3^) (Pillarisetti et al., 2017). At each study site, community air pollution (PM_1_, PM_2.5_, PM_10_, carbon monoxide, ozone, and nitrogen oxides) was measured on the roof top of a building in a fixed central location away from any proximal sources of pollution throughout the individual-level data collection periods for each study site, using two tailor-made research instruments (NAS-AF100; Sapiens Environmental Technology, Hong Kong, China).

Details of quality control and device calibration and validation have been described previously (see also Text S3 for details) (Chan et al., 2021). In brief, all devices were factory-calibrated against wood smoke by the manufacturers, and the two NAS-AF100 and nine (about 10 % of all) randomly selected PATS were further calibrated and validated via 24-h co-location with filter-based and time-resolved personal and static samplers (DustTrak DRX 8533 [TSI, MN, USA], MicroPEM [RTI International, NC, USA], and Mini-Vol portable sampler [Airmetrics, OR, USA]) for PM measurements, following standardised procedures described previously (Cao et al., 2005; Chan et al., 2021; Tong et al., 2018). Fifteen randomly selected PATS were also tested for consistency through co-location comparison tests in controlled settings for 24 h, with good agreement demonstrated (correlation coefficients: 0.85–0.99). Before and after each deployment, the PATS devices were calibrated against HEPA-filtered air for 10 min, following the manufacturer's standardised procedures.

#### 2.3.2 Data cleaning and processing

**Fig. 1:**
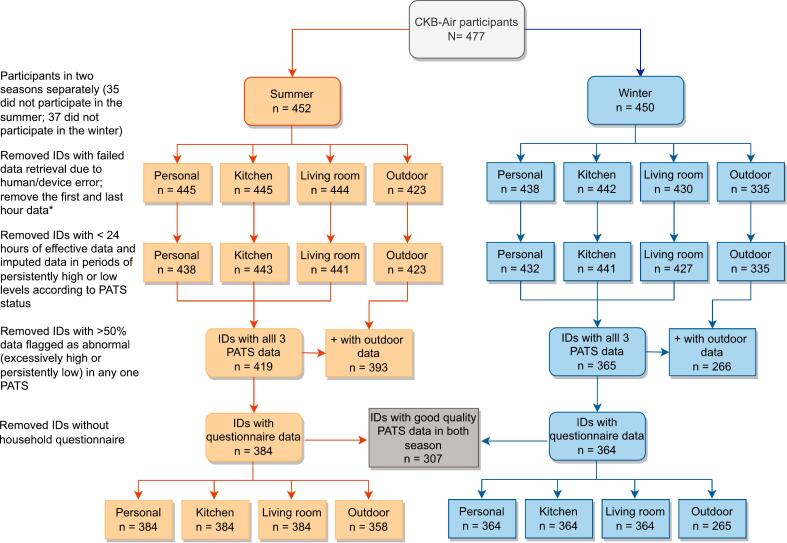
Flowchart of PATS data exclusion by season and device location. *To remove data likely to be influenced by the initial device deployment and final device collection work.

Participants with corrupted data files due to human or device error were excluded from the PM_2.5_ analyses (Fig. 1). Twenty-nine and 115 participants in the warm and cool seasons, respectively, had no community air pollution data from NAS-F100 due to delays in deployment or other logistical challenges (Fig. 1). The time-resolved PM_2.5_ data from each PATS and NAS-F100 were then inspected and processed by i) downsizing to 5-min moving averages time-series to facilitate computation, ii) applying 20-min moving median smoothing to replace sporadic extreme spikes, and iii) adjusting data points at persistently high or low (i.e. at the lower limit of detection of 10 μg/m^3^) levels by cross-device calibration. Specifically, the persistently high or low data from one device were replaced by imputed data based on levels recorded in the other devices using generalised linear regression. The persistently high levels were likely caused by particles lodged inside the nephelometer or sustained direct light impact; whereas the persistently low levels, most of which were found in the personal PATS data during winter, are likely due to an obstructed air inlet (e.g. covered by clothing) or that the PATS was placed inside an enclosed environment (e.g. in a drawer when participants took it off during bathing or sleep). Overall, only <5 % of the raw PM_2.5_ data recorded were flagged as persistently high or low, indicating generally high data quality (eTable 1).

Taking a conservative approach, we first removed data from the first and last hour of the measurement period when participants' behaviour (and hence exposure) was likely affected by the household visits and study procedures. We then removed participants with <24 h of effective data, which could happen due to battery failure. Subsequently, each of the remaining participants had at least 24 h' worth of data per PATS per season (median_summer_ [Q1-Q3]: 117 [105–119] hours, median_winter_ [Q1-Q3]: 113 [94–117] hours) (eTable 2). To further enhance the quality of the analytical dataset, we undertook a conservative approach to remove participants (n = 36) with >50 % data flagged as persistently high or low in any one PATS (regardless of the quality of the other two device) and remove participants with missing PATS data at any one location, thereby restricting the analyses to participants with the same amount of data of satisfactory quality (after imputation) across all three PATSs (n_summer_ = 419 [92.7 %]; n_winter_ = 365 [81.1 %]). Thirty-five participants in summer who did not provide household questionnaire data were further excluded. For heating fuel related analyses, two participants who reported un-specified “other” fuel type were further excluded.

The numbers of participants excluded at each stage of data analysis are shown in Fig. 1. After data cleaning, the primary analyses on PM_2.5_ included 384 and 364 participants in the summer and winter, respectively (307 participated in both seasons), with a total of 80,980 person-hours of PM_2.5_ data each at the personal, kitchen, and living room, and 67,326 person-hours of data at the community level (eTable 2). The analytical dataset contains only 483 person-hours of imputed data, all of which were in the winter subset.

### 2.4 Data analysis

Using linear regression, we estimated season-specific age- and sex-adjusted (where appropriate) geometric means and 95 % confidence intervals (CI) of personal and household (kitchen and living room) PM_2.5_ concentrations levels, by demographic characteristics (age, sex, study area, education, occupation, active/passive smoking, household size) and household air pollution-related exposures (cooking frequency, self-reported ‘smoky home’ while cooking or heating, and cooking and heating fuel combinations). For each level of microenvironment (personal, kitchen, living room, community), we aligned the time-series data (time-resolution: one reading per 5 min) for up to five 24-hour periods (totalling 120 h) during each measurement campaign and took an average across up to five values for each 5-min period from 00:00 to 24:00 for each participant, and estimated adjusted means across participants by cooking and heating fuel use categories by season.

We obtained regional temperature data during 2005–2017 (corresponding to the follow-up period of the CKB cohort up till the commencement of CKB-Air) from local meteorological offices and calculated the proportion of months with an average temperature < 10 °C in each region (0.25 for Suzhou, 0.42 for Gansu, 0.33 for Henan), which was used as a weighting coefficient to approximate heating fuel usage. We then estimated microenvironment-specific annual PM_2.5_ exposure levels as a weighted average of exposure levels across summer and winter, by cooking and heating fuel combinations: ${\mathrm{annual average}}_{\mathrm{ij}}=w_{\mathrm{ij}}*\left(1-p_{k}\right)+c_{\mathrm{ij}}*p_{k}$, where *w*_*ij*_ is the summer average and *c*_*ij*_ the winter average for microenvironment *i* (personal, kitchen, living room, or community) among participants in category *j* of cooking and heating fuel combination (no cooking or heating, clean fuels only, any solid fuels), and *p*_*k*_ is a region-specific weighting coefficient of heating fuel usage described above. These analyses were restricted to 307 participants with exposure assessment in both summer and winter.

As a preliminary investigation to understand the relationships between PM_2.5_ levels across microenvironments and by season, we examined the season-specific Spearman correlation of log-transformed PM_2.5_ levels across the four microenvironments overall and by cooking and heating fuel combinations.

### 2.5 Role of the funding source

The study funders had no role in study design, data collection, analysis, interpretation, or writing of the report. KHC, XX, KH, KBHL, and ZC had access to all data and had final responsibility for the decision to submit for publication.

## 3 Results

### 3.1 Basic characteristics and PM_2.5_ levels

Of the 384 participants included in the main analyses on the summer data, the mean age was 58.2 [SD 6.6] years, 74.7 % were women, 64.8 % were rural residents, 14.6 % were current smokers, 47.9 % were exposed to passive smoking, and 34.1 % and 52.2 % used solid fuels for cooking and heating, respectively. Those who used solid fuels for cooking were more likely to be women, from rural areas, less educated, agricultural workers or home-makers, and to use solid fuel for heating (eTable 3a). Moreover, substantially more solid fuel users reported observing a smoky home while cooking or heating compared to clean fuel users. Participants included in the analyses on the winter data (n = 364) had similar characteristics (eTable 3b).

Overall, levels of personal and household exposure to PM_2.5_ were generally higher in younger (<65 years) participants, women, rural residents, and those with lower education, with markedly higher levels in winter than in summer (Table 1). Agricultural workers, active smokers, and those exposed to passive smoking exposure and smoky homes while cooking exhibited higher PM_2.5_ exposure, most notably at personal and kitchen levels, both in summer and winter. For example, during the summer, participants observing smoky home while cooking (n = 120) had an average kitchen PM_2.5_ of 53.7 [95 % CI 49.7–58.0] μg/m^3^, whereas those without such observation had 40.9 [38.5–43.5] μg/m^3^. In winter, the contrast was even larger, being 119.5 [107.7–132.5] μg/m^3^ compared to 61.8 [56.6–67.6] μg/m^3^. Participants who reported smoky home while heating in winter (n_summer_ = 85; n_winter_ = 101) had somewhat lower personal and living room PM_2.5_ levels in summer, but significantly higher personal (82.0 [74.9–89.7] vs 55.3 [51.2–59.8] μg/m^3^), kitchen (127.9 [114.8–142.4] vs 72.6 [66.2–79.6] μg/m^3^), and living room (72.5 [66.4–79.2] vs 54.2 [50.2–58.4] μg/m^3^) levels in winter. There was no clear pattern by household size. For community PM_2.5_, younger (<65 years) participants, women, and those with higher education had consistently higher exposure across both seasons. In contrast, urban residents and factory workers had higher exposure in the summer and lower in the winter than rural residents and agricultural workers, respectively (eTable 4).

**Table 1:** Age- and sex-adjusted 24-hour geometric mean (95 % CI) PM_2.5_ concentrations (μg/m^3^) recorded in the personal, kitchen, and living room monitors by season and key characteristics.

Characteristics	Summer (N⁎ = 384)				Winter (N⁎ = 364)			
	N⁎	Personal	Kitchen	Living room	N⁎	Personal	Kitchen	Living room
Personal characteristics								
*Age*†								
< 65 years	323	42.0 (40.2–43.9)	43.9 (41.8–46.2)	33.5 (32.1–34.9)	305	59.5 (56.2–63.1)	82.8 (77.2–88.8)	56.1 (52.9–59.4)
≥ 65 years	61	33.5 (30.7–36.5)	43.0 (39.0–47.5)	31.9 (29.4–34.6)	59	48.9 (43.6–54.9)	62.9 (54.7–72.2)	49.2 (43.9–55.1)
*Sex*‡								
Female	287	40.9 (39.3–42.6)	44.4 (42.4–46.5)	32.7 (31.4–33.9)	267	66.1 (62.6–69.8)	87.6 (82.1–93.5)	61.1 (57.9–64.5)
Male	97	39.8 (37.2–42.7)	43.3 (40.1–46.9)	33.7 (31.6–36.0)	97	50.2 (45.8–55.0)	72.0 (64.5–80.4)	49.4 (45.1–54.1)
*Region*								
Rural	249	36.3 (34.7–38.0)	53.4 (50.7–56.3)	48.9 (46.7–51.1)	251	66.5 (62.7–70.7)	107.7 (100.3–115.6)	74.5 (70.2–79.0)
Urban	135	28.2 (26.6–29.8)	30.7 (28.7–32.8)	28.6 (26.9–30.3)	113	36.8 (33.9–40.0)	42.1 (38.1–46.5)	33.7 (31.0–36.6)
*Education*								
No formal education	97	48.9 (45.1–53.1)	52.3 (47.6–57.5)	38.2 (35.3–41.3)	92	75.8 (67.9–84.7)	112.6 (98.7–128.5)	70.2 (62.9–78.3)
Primary & middle school	133	38.8 (36.5–41.4)	42.8 (39.8–46.0)	30.4 (28.7–32.3)	125	58.5 (53.8–63.5)	84.0 (76.1–92.8)	58.4 (53.8–63.4)
High school or above	154	38.4 (36.3–40.7)	41.6 (39.0–44.4)	33.3 (31.5–35.1)	147	50.9 (47.1–55.0)	66.0 (60.2–72.4)	47.4 (44.0–51.2)
*Occupation*								
Agricultural worker	140	53.2 (50.3–56.3)	59.9 (56.2–63.9)	38.0 (36.0–40.2)	138	74.6 (69.2–80.4)	120.0 (109.6–131.3)	69.0 (63.9–74.4)
Factory worker	20	34.4 (29.7–39.9)	39.1 (33.0–46.2)	31.7 (27.4–36.6)	18	59.0 (47.9–72.7)	59.2 (46.0–76.1)	36.9 (29.9–45.5)
Home-maker	106	40.1 (37.3–43.2)	43.2 (39.7–47.0)	32.9 (30.6–35.4)	109	68.6 (62.3–75.5)	88.4 (78.8–99.2)	65.3 (59.3–72.0)
Non-manual labour	9	33.4 (27.0–41.4)	29.0 (22.7–37.0)	26.2 (21.2–32.4)	10	62.0 (47.1–81.8)	46.0 (33.0–64.2)	45.2 (34.2–59.7)
Self/un-employed or other	109	29.1 (27.2–31.1)	30.7 (28.4–33.1)	28.5 (26.7–30.5)	89	31.6 (28.7–34.8)	43.7 (39.0–49.1)	37.4 (33.9–41.2)
*Current active smoker*								
No	328	38.6 (36.5–40.8)	44.7 (42.0–47.6)	29.2 (27.7–30.8)	303	52.1 (48.2–56.3)	75.0 (68.3–82.3)	49.1 (45.5–53.0)
Yes	56	45.1 (40.7–50.1)	41.9 (37.2–47.2)	45.6 (41.3–50.3)	61	71.9 (62.7–82.4)	90.2 (76.5–106.3)	70.5 (61.6–80.7)
*Passive smoking exposure*§								
No	200	38.7 (36.7–40.9)	42.9 (40.4–45.6)	30.6 (29.1–32.2)	180	50.7 (47.1–54.6)	51.8 (48.1–55.7)	75.9 (69.5–83.0)
Yes	184	42.0 (39.9–44.3)	44.8 (42.2–47.6)	35.8 (34.1–37.7	184	64.0 (59.8–68.5)	57.7 (53.9–61.7)	82.4 (75.9–89.5)
*Household size*⁎⁎								
≤ 4 persons	196	39.6 (37.7–41.7)	42.6 (40.2–45.2)	34.2 (32.6–35.9)	190	55.5 (51.8–59.5)	78.9 (72.6–85.8)	55.9 (52.2–59.9)
> 4 persons	188	41.2 (39.0–43.5)	45.3 (42.6–48.1)	32.0 (30.5–33.7)	174	60.0 (55.8–64.5)	79.9 (73.3–87.2)	53.8 (50.1–57.8)
*Cooking frequency*								
Daily personal cooking	301	41.4 (39.2–43.8)	43.6 (41.0–46.5)	34.4 (32.6–36.3)	280	59.2 (54.7–64.1)	82.4 (74.9–90.6)	55.3 (51.1–59.8)
Daily household cooking	41	37.6 (33.7–42.1)	49.2 (43.4–55.9)	31.3 (28.2–34.8)	44	52.1 (44.9–60.4)	75.2 (63.0–89.8)	54.6 (47.2–63.2)
Infrequent cooking	42	39.6 (35.6–44.1)	39.9 (35.3–45.1)	30.8 (27.8–34.1)	40	58.3 (50.4–67.5)	74.5 (62.5–88.7)	54.1 (46.9–62.5)
*Smoky home while cooking*††								
No	222	37.2 (35.2–39.2)	40.9 (38.5–43.5)	32.4 (30.8–34.1)	196	45.7 (42.4–49.1)	61.8 (56.6–67.6)	46.8 (43.4–50.3)
Yes	120	46.1 (43.0–49.3)	53.7 (49.7–58.0)	35.1 (32.9–37.4)	127	74.9 (68.7–81.6)	119.5 (107.7–132.5)	67.7 (62.1–73.8)
*Smoky home while heating*‡‡								
No	163	44.3 (41.8–47.0)	44.1 (41.3–47.2)	34.0 (32.1–35.9)	151	55.3 (51.2–59.8)	72.6 (66.2–79.6)	54.2 (50.2–58.4)
Yes	85	39.9 (36.8–43.1)	51.4 (46.9–56.4)	29.5 (27.4–31.8)	101	82.0 (74.9–89.7)	127.9 (114.8–142.4)	72.5 (66.4–79.2)

⁎ Number of subjects.

† Only adjusted for sex. The age cut-off was set to separate middle-aged adults and elderly following the convention.

‡ Only adjusted for age.

§ Total passive smoking exposure (frequency per week) of >3–5 days/week was defined as exposed (“Yes”).

⁎⁎ The 4-person household size cut off was set on the median of the reported household size in the sample.

†† Restricted to individuals who reported frequent household or personal cooking only.

‡‡ Restricted to individuals who reported heating in winter only.

### 3.2 PM_2.5_ levels by fuel use patterns

**Fig. 2:**
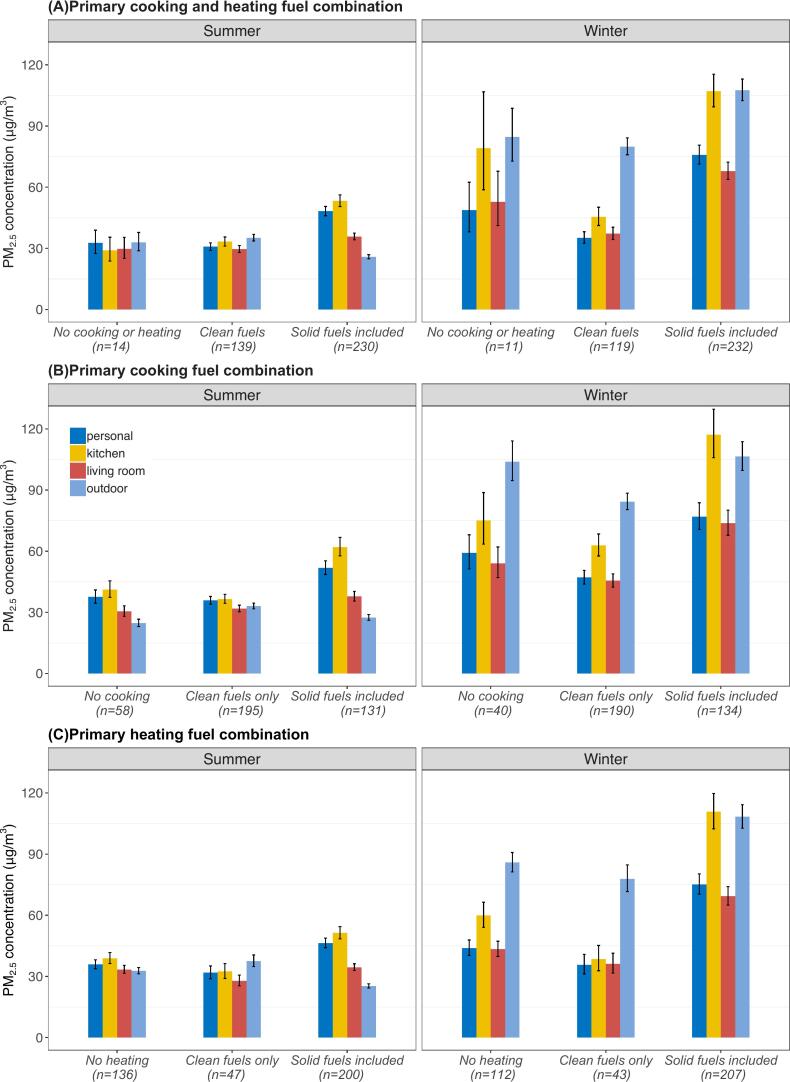
Age- and sex-adjusted 24-hour geometric mean PM_2.5_ concentrations (μg/m^3^) recorded in the personal, kitchen, living room, and community monitors by season and the combination of primary cooking and heating fuels. Each vertical bar represents adjusted geometric means of each microenvironment by exposure groups, with vertical black lines showing the corresponding 95 % confidence intervals (CIs). Non-overlapping CIs between bars indicate statistically significant difference. From left to right the four bars in each group are personal, kitchen, living room, and community PM_2.5_ levels. Participants reporting using unspecified “other” fuels for heating were excluded due to small sample size (N_summer_ = 1; N_winter_ = 2).

Compared across different combinations of primary cooking and heating fuels, solid fuel users had higher personal and kitchen PM_2.5_ levels than those who used clean fuels (personal: by 57 % in summer and 115 % in winter; kitchen: by 60 % in summer and 136 % in winter) and those who did not cook or heat (personal: 48 % in summer and 55 % in winter; kitchen: 83 % in summer and 35 % in winter), respectively (Fig. 2A). In winter, solid fuel users had significantly (57–101 %) higher personal and household PM_2.5_ levels, whereas the increase was less pronounced for clean fuel users (14–37 %). In contrast, community levels were consistently 2–4 times higher across different fuel combinations. Broadly similar patterns were observed when examining cooking and heating fuel combinations separately (Fig. 2B and C). Sensitivity analyses restricting to participants who cooked regularly by themselves (eFigure 2) and excluding participants with any imputed data (eFigure 3) showed similar patterns.

Consistently, participants who had used solid fuels for cooking or heating had the highest weighted average annual PM_2.5_ exposure at the personal (78.2 [71.6–85.3] μg/m^3^; 70 % higher), kitchen (102.4 [90.4–116.0] μg/m^3^; 84 % higher), and living room (62.1 [57.3–67.3] μg/m^3^; 50 % higher) microenvironments, compared to those who reported using clean fuels or not cooking or heating (Table 2). There was no statistically significant difference in annual community PM_2.5_ levels across these groups, although solid fuel users tended to have slightly higher exposure. Similar patterns were observed when examining cooking and heating fuel combinations separately. Additional adjustment for active and passive smoking did not alter the findings meaningfully (eTable 5).

**Table 2:** Age- and sex-adjusted estimated annual 24-hour geometric mean PM_2·5_ exposure levels (μg/m^3^) for the personal, kitchen, living room, and community environments by cooking and heating fuel category.

Cooking and heating fuel category	Personal	Kitchen	Living room	Community
Primary cooking fuel combination				
No cooking (n = 45)	60.0 (50.6–71.2)	85.5 (67.8–107.7)	51.5 (44.4–59.8)	53.8 (46.4–62.4)
Clean fuels only (n = 155)	50.7 (45.7–56.3)	59.7 (51.9–68.7)	45.6 (41.6–49.9)	56.0 (52.1–60.2)
Solid fuels included (n = 107)	84.9 (74.9–96.3)	116.8 (98.6–138.4)	68.3 (61.2–76.2)	63.9 (57.9–70.5)
Primary heating fuel combination				
No heating (n = 98)	50.2 (44.4–56.8)	65.9 (55.7–77.9)	47.2 (42.4–52.7)	56.3 (51.8–61.2)
Clean fuels only (n = 39)	43.4 (36.1–52.2)	44.5 (34.6–57.2)	39.3 (33.4–46.3)	53.7 (47.7–60.6)
Solid fuels included (n = 169)	76.3 (69.2–84.2)	103.5 (90.6–118.3)	61.3 (56.3–66.9)	61.4 (56.3–66.9)
Primary cooking and heating fuel combination				
No cooking or heating (n = 8)	50.2 (34.3–73.6)	59.2 (34.4–101.8)	41.7 (29.4–59.0)	52.0 (40.5–66.8)
Clean fuels (n = 107)	41.6 (37.3–46.5)	52.3 (44.8–61.2)	41.0 (37.1–45.3)	54.2 (50.2–58.4)
Solid fuels included (n = 191)	78.2 (71.6–85.3)	102.4 (90.4–116.0)	62.1 (57.3–67.3)	62.1 (57.4–67.2)

Annual mean level was estimated using the regional temperature data from 2005 to 2017, the number of months with average temperature < 10 degrees are 3/12 in Suzhou, 5/12 in Gansu, 4/12 in Henan. Using the same data, the proportion of days with ≤10 degrees daily average temperature are 1233/4717 (26.1 %) in Suzhou, 1921/4717 (40.7 %) in Gansu, 1524/4717 (32.7 %) in Henan. The analyses were restricted to participants with good quality PM_2.5_ data in both seasons (n = 307). The analyses for were restricted to 307 participants with repeated measurements in both summer and winter, and one participant reporting unspecified “other” fuel for cooking was further excluded from the heating-related analyses.

**Fig. 3:**
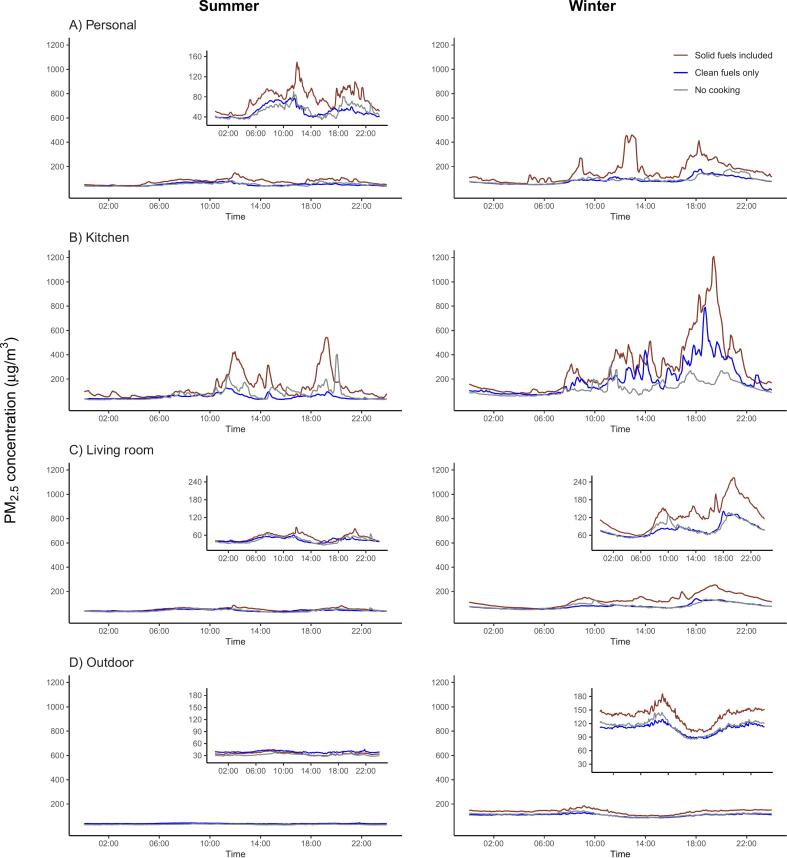
24-hour average time-series plots for PM_2.5_ concentrations (μg/m^3^) recorded in the personal, kitchen, living room, and community monitors by season and primary cooking fuel combinations. There were 123 (13,761 person-hour), 167 (18,663 person-hour) and 94 (10,607 person-hour) subjects for the “Solid fuels included”, “Clean fuels” and “No cooking” group in summer, respectively; There were 126 (13,174 person-hour), 173 (17,956 person-hour) and 65 (6819 person-hour) subjects for the “Solid fuels included”, “Clean fuels” and “No cooking” group in winter, respectively. Smaller plots nested within panels are “zoom-in” version of the corresponding plot, as the use of a universal y-axis limit up to 1200 with reference to the kitchen exposure levels impaired the readability of those plots.

**Fig. 4:**
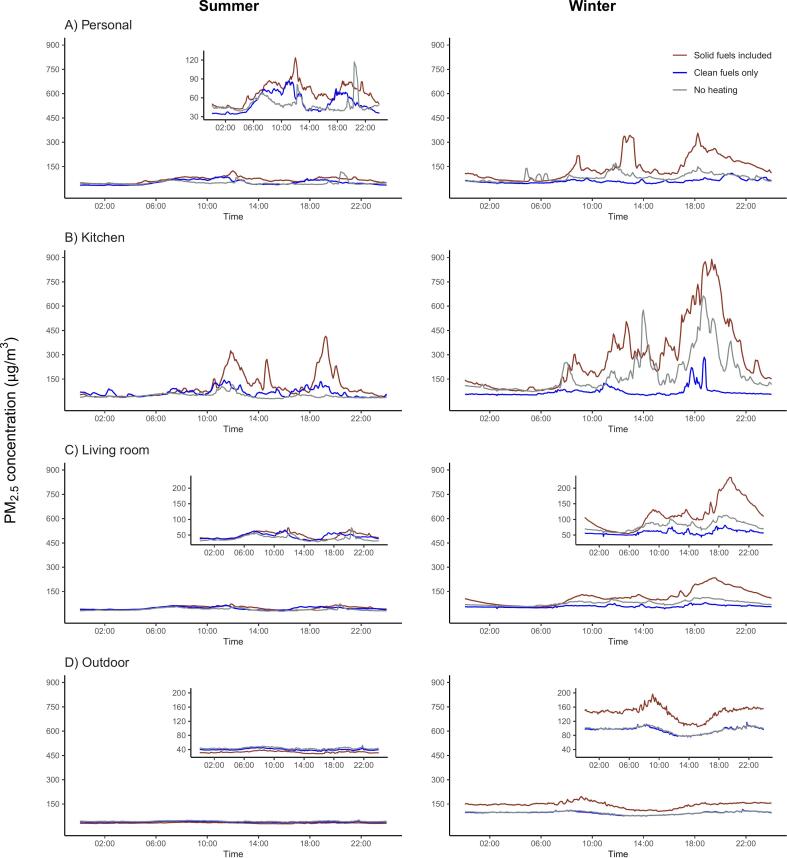
24-hour average time-series plots for PM_2.5_ concentrations (μg/m^3^) recorded in the personal, kitchen, living room, and community monitors by season and primary heating fuel combinations. There were 200 (22,539 person-hours), 47 (5226 person-hours) and 136 (15,147 person-hours) subjects for the “Solid fuels included”, “Clean only” and “No heating” group in summer, respectively; There were 207 (21,772 person-hours), 43 (4261 person-hours) and 112 (11,701 person-hours) subjects for the “Solid fuels included”, “Clean only” and “No heating” group in winter, respectively. Smaller plots nested within panels are “zoom-in” version of the corresponding plot, as the use of a universal y-axis limit up to 1200 with reference to the kitchen exposure levels impaired the readability of those plots.

When inspecting the aggregated diurnal PM_2.5_ levels visually, we observed major peaks at around noon and evening time for both solid fuel and clean fuel users, and these peaks were substantially higher in solid fuel users, up to ~500 and ~1200 μg/m^3^ in summer and winter, respectively (Fig. 3A-C). A small morning peak (~08:00) was also found in the kitchen in winter (Fig. 3B). Solid fuel users appeared to have lower community PM_2.5_ exposure than clean fuel users in summer, but have the highest levels in winter, with broadly concordant diurnal variations for all three fuel use categories in both seasons (Fig. 3D). The differences in diurnal exposure levels by heating fuel combinations were largely similar to those by cooking fuels in summer, but in winter the exposure levels in individuals who did not have heating were just slightly lower than the solid fuel users but much higher than clean fuel users, especially in kitchens (Fig. 4).

### 3.3 PM_2.5_ exposure models and inter-spatial correlation

**Fig. 5:**
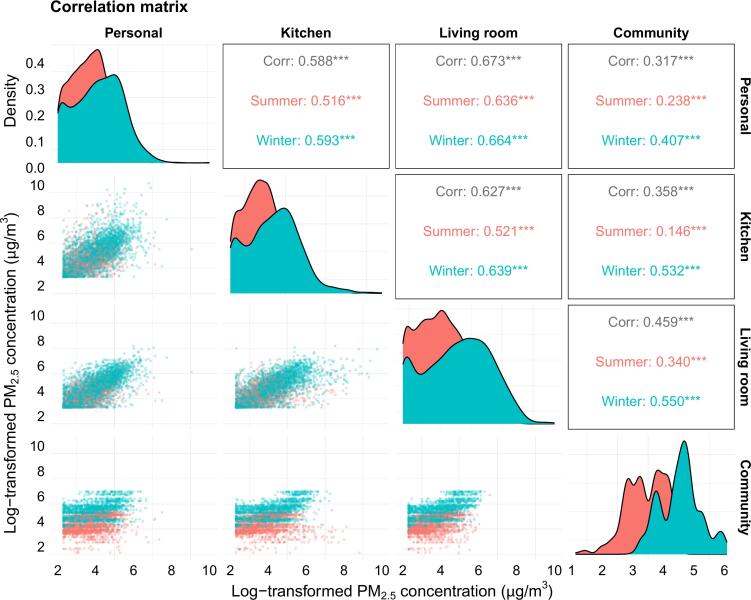
The correlation matrix between the log-transformed concentrations of PM_2.5_ at personal, kitchen, living room and community levels. Red area under curves and dots are summer data; blue area under curves and dots are winter data; black numbers in boxes are overall Spearman correlation coefficient; red and blue numbers are summer- and winter-specific correlation.

We found moderate correlation between personal and household PM_2.5_ levels (living room Spearman's r: 0.64–0.66; kitchen r: 0.52–0.59). In contrast, the correlation between community levels and personal or household levels was weaker, especially in summer, with Spearman's r ranging 0.15–0.34 in summer and 0.41–0.55 in winter (Fig. 5). Among the different cooking and heating fuel combinations, the correlation between community levels and personal or household levels was the strongest in those reporting no cooking or heating, with Spearman's r ranging 0.47–0.68 in summer and 0.43–0.46 in winter, and the weakest in those who had used solid fuels, with the corresponding range of 0.11–0.31 and 0.29–0.52, respectively (eFigures 4A-C).

## 4 Discussion

We reported integrated and time-resolved PM_2.5_ levels at personal, household (kitchen and living room), and community environments by cooking and heating fuel combinations and other key characteristics in 384 adults in summer and 364 adults in winter from one urban and two rural areas of China. Solid fuel use for cooking and heating was associated with significantly higher estimated annual PM_2.5_ exposure at both personal and household levels: personal PM_2.5_ exposure in summer and winter was over 3 times and 5 times the World Health Organization 24-hour Air Quality Guidelines (WHO AQG) level (15 μg/m^3^), respectively and the annual personal exposure was over 15 times of the WHO annual AQG level (5 μg/m^3^) (*WHO Global Air Quality Guidelines. Particulate Matter (PM2.5 and PM10), Ozone, Nitrogen Dioxide, Sulfur Dioxide and Carbon Monoxide*, 2021). The PM_2.5_ levels across all microenvironments were higher in winter than summer, with about 2–4 times higher community levels across fuel categories. Time-resolved data showed vast inter- and intra-personal variability in PM_2.5_ exposure within and across seasons, with remarkably high exposure (5-min moving-average up to 1200 μg/m^3^) recorded in typical cooking times (about 2–4 h per day) among solid fuel users, especially in the kitchen.

Previous studies assessing exposures to air pollution were highly heterogeneous in settings, sample size, prevalent fuel types, and recorded PM_2.5_ levels (Balakrishnan et al., 2018; Benka-Coker et al., 2020; Ni et al., 2016; Shupler et al., 2018; Shupler et al., 2020; Sidhu et al., 2017; Snider et al., 2018; Tong et al., 2018; Ye et al., 2020), but there has been broadly consistent evidence that solid fuel use for cooking was associated with higher personal and kitchen PM_2.5_ levels as reported in our study. For logistical and technical reasons, most previous studies primarily measured kitchen PM_2.5 (__Balakrishnan et al., 2018__;_ _Benka-Coker et al., 2020__;_ _Han et al., 2021__;_ _Ni et al., 2016__;_ _Shupler et al., 2018__;_ _Shupler et al., 2020__;_ _Sidhu et al., 2017__;_ _Snider et al., 2018__;_ _Tong et al., 2018__;_ _Ye et al., 2020__)_, while some had parallel measurements of personal (Ni et al., 2016; Shupler et al., 2020; Ye et al., 2020) or ambient (Han et al., 2021; Ni et al., 2016; Snider et al., 2018) exposure, with most personal measurements done in a subset of participants. Notably, the largest single sample (n = 910; ~48,000 person-hours) of personal PM_2.5_ measurements (alongside kitchen measurements in 2365 households) came from the PURE-Air study focussing on cooking fuel in rural areas across eight countries (Shupler et al., 2020). With 48-hour integrated PM_2.5_ measurements, they found lower PM_2.5_ levels by cooking fuel types moving up the traditional ‘energy ladder’ (i.e. from heavily polluting biomass to coal, then to gas and electricity) (Gordon et al., 2014), but they also found substantial heterogeneity within each solid fuel category and between countries (e.g. kitchen PM_2.5_ for primary wood use was 50 [45–55] μg/m^3^ in China and 105 [96–116] μg/m^3^ in India), possibly due to varying fuel use behaviour or infrastructure, chemical constituents of fuels, and different climate conditions (Shupler et al., 2020). With the parallel and repeated time-resolved assessment of personal, kitchen, living room (80,980 person-hours for each measure), and community (67,326 person-hours) PM_2.5_ levels in summer and winter (eTable 2), we provided further insight into the complex relationships between fuel use behaviour and PM_2.5_ levels across the personal-household-community exposure spectrum.

Consistent with the growing number of studies that ascertained multiple fuel use (Ni et al., 2016; Shupler et al., 2020; Snider et al., 2018), we showed that fuel stacking was common in rural China, and even mixed use of solid and clean fuels was associated with substantially elevated PM_2.5_ exposure, especially in winter. As fuel stacking is increasingly common in many developing economies, this highlights the importance of capturing usage information beyond a single, primary fuel type, in order to more accurately assess household air pollution exposure and the associated disease burden. On the other hand, before 2015, heating has not commonly been considered as a major contributor of air pollution by researchers and policymakers (Chan et al., 2017; Chen et al., 2018; Tao et al., 2018; World Health Organization, 2016). Our findings add to the growing field measurement evidence (Carter et al., 2016; Liao et al., 2017; Walker et al., 2021), showing those who used solid fuels for heating to have 91–188 % higher personal and household PM_2.5_ exposure in winter, when compared to clean fuel users. It may seem counterintuitive to observe a higher level in the kitchen than in the living room, but previous studies have noted generally poorer ventilation in winter and that solid fuel users may stay in the kitchen longer to get warmth from the cookstove to save fuel (Gordon et al., 2014). In fact, we have previously reported a slower rate of modernisation of heating (versus cooking) fuel in China (as in many other LMICs) (Chan et al., 2017). Adding to the complexity, the lack of heating in rural China was associated with a lower socioeconomic status and greater likelihood of using solid fuels for cooking compared to clean fuel users (Chan et al., 2017). This, together with the likely reduced ventilation (to keep warm) in winter time, may explain the considerably (25 %) higher personal and household PM_2.5_ levels (in both seasons) among our participants who reported ‘no heating’, compared with the clean heating fuel users.

While the community PM_2.5_ level was markedly higher in winter in both solid (about 4 times) and clean (about 2 times) fuel users, there was an interesting contrast that solid fuel users had lower community PM_2.5_ than clean fuel users in summer, but higher in winter. The ‘winter smog’ phenomenon in densely populated (often urban) areas of China is well-documented, as increased energy consumption, reliance on coal-fired power plants, and meteorological factors (e.g. temperature inversion) drive heightened regional ambient air pollution, while the limited stringent environmental regulations or poor enforcement of such are relevant contextual factors (Gu and Yim, 2016). On the other hand, most solid fuel users resided in rural areas with lower population and vehicle density, which tend to be associated with lower ambient air pollution. In winter, however, the intensive use of solid fuels for heating (most participants reported heating throughout the day) could result in major rise of neighbourhood PM_2.5_ in addition to regional ambient air pollution, as supported by previous studies (Hu et al., 2020; Ni et al., 2016). It is also worth noting that the increase in personal and household levels across seasons was much higher in solid fuel users than in clean fuel users, whose personal and household levels were <50 % of the community levels.

As in numerous previous studies (Han et al., 2021; Ni et al., 2016) we observed relatively weak correlation between personal and community PM_2.5_ levels. This poses questions to the commonly adopted exposure assessment methods in previous epidemiological studies, particularly modelled ambient PM_2.5_ levels, often applied without accounting for inter-spatial variability and people's time spent indoors (typically 70–80 %) (Murray et al., 2020). This may be less problematic in HICs with relatively low exposure from non-ambient sources, although the re-emergence of wood-fire heating may raise concern. The relatively strong correlation (0.52–0.66) between personal and household measurements is consistent with previous evidence (e.g. PURE-Air: person-to-kitchen correlation = 0.69) (Shupler et al., 2020). Our evidence adds further support for more accurate personal exposure estimation via household measurements along with housing characteristics questionnaires, simple personal GPS trackers, and advanced ambient air pollution modelling approaches (Li et al., 2020; Schneider et al., 2020), especially in large-scale epidemiological studies where extensive personal measurement is infeasible. More in-depth modelling analysis on our data will generate further insight for better exposure approximation in future studies.

Our time-resolved data also illustrated the remarkable short-term intra- and inter-personal variability in PM_2.5_ exposure even within each fuel use category. The diurnal patterns of kitchen PM_2.5_ appeared consistent with the previously reported time-activity patterns in CKB-Air (Chan et al., 2021), such as the exposure peaks (averaged twice in summer; 3 times in winter) at typical meal times. Furthermore, we observed stronger and longer-lasting evening peaks of personal and household levels among individuals who used solid fuels in winter, which is consistent with typical space heating practices with reduced ventilation at night. The vast diurnal variations, with personal PM_2.5_ exposure as high as 400 μg/m^3^ and as low as 10 μg/m^3^, lead to the question of whether and how long-term average exposure could compare to an accumulation of repeated bursts of extreme exposure on a time scale of minutes to hours compare to longer-term average exposure (e.g. between day, seasonal, or annual) in relation to disease development risk (Smith and Kriebel, 2013). Conventional epidemiological approaches examine short- (days) and long- (years) term exposure-outcome associations separately with limited consideration on their interplay. Questions remain as to the extent to which environmental risk factors increase disease risk by causing gradual pathophysiological changes versus repeated acute events triggering clinically significant discomfort or illnesses. The mystery might be solved by the increasing availability of more refined air pollution data, the use of chamber studies, and the emerging multi-omics technologies that facilitate a better understanding of the toxicology and pathophysiology.

CKB-Air offers one of the most detailed parallel and repeated seasonal assessments of personal, household, and community level PM_2.5_ with one of the largest time-resolved datasets (67,326–80,980 person-hours per microenvironment). Moreover, we assessed not only the role of parallel fuel use for cooking but also for heating on both average and time-resolved PM_2.5_ exposure, shedding light on the complexity of fuel use behaviour and PM_2.5_ exposure. However, several limitations warrant discussion. First, despite the relatively large amount (in person-hour) of data captured, the number of participants representing each fuel use combination beyond the aggregated categories on solid versus clean fuels was small. Also, the large inter-and intra-personal variability means that we could not reliably estimate PM_2.5_ levels by >10 different fuel combinations captured. Moreover, as the majority of clean fuel users reported mixed use of gas and electricity, and the solid fuel users reported mixed use of solid and clean fuels, the present study was not suited to assess PM_2.5_ levels associated individual fuel types in population with high level of fuel-stacking. Second, unlike some previous studies that used gold-standard gravimetric samplers in measuring integrated PM_2.5_ exposure (Shupler et al., 2020), we used a nephelometer in order to obtain detailed time-resolved data. Despite the field- and lab-based validation and calibration, our instruments inevitably entailed random measurement error and, potentially, over-estimation at very high levels of relative humidity (>90 %) or PM_2.5_ (>1000 μg/m^3^), a common challenge of nephelometer-based devices. Designed for high-pollution settings, the built-in low- and high-channel algorithm of PATS and the regular wood smoke and zeroing calibration should reduce (but not eliminate) these errors. Third, we assessed community PM_2.5_ at a single location, and we lacked pairwise data of street and regional levels. Fourth, the study sample was recruited via convenient sampling from three purposively selected areas in China, so the estimated exposure levels would not be generalisable to China or other populations. Similarly, given the stark contrast in fuel use patterns between the rural and urban sites, we could not conduct reliable subgroup analysis by urbanity. Fifth, the household questionnaire on participant characteristics was only administered in winter. Although we assessed most characteristics (e.g. cooking frequency) on a 12-month time frame, participants had to recall their cooking fuel types used in summer if these differed in winter, so recall bias may exist.

## 5 Conclusions

This study has demonstrated the feasibility and value of collecting detailed air pollution exposure measurement data to capture intra- and inter-personal variations over short (weekly) and medium (seasonal) term, in rural and urban China. Most notably, the individuals who used solid fuels for cooking or heating were found to have annual personal PM_2.5_ exposure over 15 times higher than the latest WHO AQG. The relatively weak correlation of personal with community PM_2.5_, in contrast to the stronger correlation between personal and household levels, supports the use of reliable, low-cost household static monitors in improving personal air pollution exposure assessment in large-scale epidemiological studies. Our findings underscores the complexity of air pollution exposure and the need for cross-disciplinary investigation involving exposure science, toxicology, epidemiology and statistics.

## Open access statement

This research was funded in whole, or in part, by the 10.13039/100010269Wellcome Trust [Grant number 212946/Z/18/Z, 202922/Z/16/Z, 104085/Z/14/Z, 088158/Z/09/Z, 223100/Z21/Z]. For the purpose of Open Access, the author has applied a CC-BY public copyright licence to any Author Accepted Manuscript version arising from this submission.

## Data access statement

The China Kadoorie Biobank (CKB) is a global resource for the investigation of lifestyle, environmental, blood biochemical and genetic factors as determinants of common diseases. The CKB study group is committed to making the cohort data available to the scientific community in China, the UK and worldwide to advance knowledge about the causes, prevention and treatment of disease. For detailed information on what data is currently available to open access users and how to apply for it, visit: http://www.ckbiobank.org/site/Data+Access.

Researchers who are interested in obtaining the raw data from the China Kadoorie Biobank study that underlines this paper should contact ckbaccess@ndph.ox.ac.uk. A research proposal will be requested to ensure that any analysis is performed by bona fide researchers.

## CRediT authorship contribution statement

**Ka Hung Chan:** Conceptualization, Methodology, Software, Formal analysis, Investigation, Resources, Data curation, Writing – original draft, Writing – review & editing, Visualization, Project administration, Supervision. **Xi Xia:** Methodology, Software, Formal analysis, Investigation, Writing – review & editing, Visualization. **Cong Liu:** Methodology, Resources, Data curation, Writing – review & editing. **Haidong Kan:** Methodology, Resources, Data curation, Writing – review & editing. **Aiden Doherty:** Methodology, Investigation, Writing – review & editing. **Steve Hung Lam Yim:** Methodology, Resources, Writing – review & editing. **Neil Wright:** Methodology, Software, Data curation, Writing – review & editing. **Christiana Kartsonaki:** Methodology, Software, Data curation, Writing – review & editing. **Xiaoming Yang:** Resources, Data curation, Software. **Rebecca Stevens:** Resources, Data curation, Software. **Xiaoyu Chang:** Resources, Project administration. **Dianjianyi Sun:** Resources, Project administration. **Canqing Yu:** Resources, Project administration, Funding acquisition. **Jun Lv:** Resources, Project administration, Funding acquisition. **Liming Li:** Resources, Project administration, Funding acquisition. **Kin-Fai Ho:** Conceptualization, Methodology, Resources, Writing – review & editing, Supervision. **Kin Bong Hubert Lam:** Conceptualization, Methodology, Resources, Writing – review & editing, Project administration, Supervision, Funding acquisition. **Zhengming Chen:** Conceptualization, Methodology, Resources, Writing – review & editing, Project administration, Supervision, Funding acquisition.

## Declaration of competing interest

The authors declare that they have no known competing financial interests or personal relationships that could have appeared to influence the work reported in this paper.

## Data Availability

See data access statement in manuscript.

## Appendix A Supplementary data

Supplementary material Image 1
